# The evolution of parasite genomes and the origins of parasitism

**DOI:** 10.1017/S0031182014001516

**Published:** 2015-02

**Authors:** ANDREW P. JACKSON

**Affiliations:** Department of Infection Biology, Institute of Infection and Global health, University of Liverpool, Ic2 Building, 146 Brownlow Hill, Merseyside, Liverpool, L3 5RF

**Keywords:** parasitism, genome, evolution

From a human perspective it seems intuitive to view life as essentially free-living, and parasites as specialized derivatives. Certainly, this is the impression given by biology teaching or the balance of research effort. In fact, it is just conceivable that parasites are more numerous than non-parasites (Dobson *et al.* 2008), and while this may be difficult to prove, they are clearly abundant and ubiquitous throughout evolutionary history (Conway Morris, 1981). Today, we appreciate the great importance parasites have for the ecology, behaviour and evolution of free-living organisms, but also for biodiversity and ecosystem function (Lafferty *et al.* 2006; Kuris *et al.* 2008; Dunne *et al.* 2013). As moderators of trophic dynamics and competition between free-living species, parasites have been called ‘ecosystem engineers’ (Hatcher *et al.* 2012) and as vital components of all ecosystems the evolutionary origins of parasites are a core issue in evolutionary biology, although it has not always been so.

In April 2013, parasitologists working on various parasites and with diverse approaches met at the Wellcome Trust Conference Centre in Hinxton (UK) for a meeting entitled ‘*The evolution of parasite genomes and the origins of parasitism*’. The purpose of the meeting was to promote consensus on the impact of genomics on the evolution of parasitism and to identify any themes in genome evolution that cut across taxonomic boundaries. Most research communities are now approaching a point where genome sequences exist for multiple species of their chosen parasite. Therefore, the question of origins and diversification are being posed in many quarters. In principle, the wealth of comparative genomic data provides an opportunity to test long-standing hypotheses of genome reduction, adaptation, the evolution of complexity, and host–parasite co-evolution with unprecedented accuracy and clarity. This special issue contains ten review articles developing the discussions that took place.

From the 19th century birth of evolutionary biology until well into the 20th century, parasites were seen as biologically degenerate, ecologically marginal and evolutionarily anomalous. The first generation of evolutionary thinkers retained a belief that evolution was progressive and tending towards perfection. Cope's 1896 Law that evolution proceeds from unspecialized to specialized forms left parasites estranged; they had specialized in regression. Whether this was a lingering sense of orthogenesis, the afterglow of a *Scala Naturae*, or an ingrained anthropocentricity, evolutionary biologists struggled to see parasites, apparently an insult to evolutionary progress, as fully fledged organisms, and considered parasitic adaptations less authentic than those of non-parasites.

Vickerman (2009) describes how this atmosphere contributed to the insularity of parasitology and its poor relationship with the study of evolution. The comment by Konrad Lorenz, as late as 1973, that ‘*If one judges the adapted forms of the parasites according to the amounts of retrogressed information, one finds a loss of information that coincides with and completely confirms the low estimation we have of them and how we feel about them*’ is worth repeating because it encapsulates the engrained view that becoming a parasite is mostly about inexorable degradation. Today, any concept of progress in evolution is based on fitness metrics that define parasites as well as any organism. While we have discarded the notions of directionality and chauvinism that prejudiced our analysis of parasitism, it casts a pall over parasite evolution and affects the questions we tend to ask.

The arrival of molecular phylogenetics made it possible to consider the origin and diversification of parasites explicitly and with sufficient power. Parasites are now a subject for evolutionary biologists, who consider their roles in the origins of sex and in speciation, while evolution is a subject for parasitologists, particular with regard to drug resistance, virulence and host range. The application of molecular phylogenetics to parasite origins brought seminal developments of the ecological–morphological narrative that dominated parasite origins previously. In 2003, a collection of articles were published in *Advances in Parasitology*, which discussed such developments in the phylogeny of *Plasmodium* (Rich and Ayala, 2003), nematodes (Blaxter, 2003) and digeneans (Cribb *et al.* 2003), as well as methods for extracting evolutionary signatures from molecular data (Drummond *et al.* 2003; McInerne *et al.* 2003). In some respects, we are seeking to update these discussions here in the light of genomics.

Genome sequences have revolutionized the study of parasite evolution. They have provided an exhaustive sample of molecular characters to improve parasite phylogenies that previously might have been based on single genes, and been poorly resolved as a consequence. The same sampling allows deep phylogenetic comparisons between taxa that are not related well on morphology or ultrastructure. For example, the robust placement of the dinoflagellates and Apicomplexa as sister clades has only been possible through plastid genome comparison (Janouskovec *et al.* 2010). Naturally, complete gene repertoires for parasites have exposed the genetic basis to their specialized phenotypes, especially with respect to cell surface-expressed proteins. In diverse clades, species-specific genes are often enriched for functions on or beyond the parasite surface, for example among trypanosomatids (El-Sayed *et al.* 2005, Jackson *et al.* 2012), *Plasmodium* spp. (Carlton *et al.* 2008), the plant pathogenic oomycetes (Tyler *et al.* 2006; Adhikari *et al.* 2013) and tapeworms (Tsai *et al.* 2013), showing that these genes evolve fastest.

Even for problems that are not explicitly phylogenetic, genome sequencing is making evolutionary thinking more prominent within parasitology because the sequencing of genomes from multiple parasite species and strains makes comparison intuitive and necessary. Comparative genomics is predicated on phylogeny and interpreted likewise, so we can hope that the wealth of easily accessed genomic resources will lead to parasite gene function being considered in a comparative context routinely, promoting an evolutionary perspective even if this is not the primary concern.

This special issue begins with an article by Poulin and Randhawa (2014) introducing the ecological paradigm within which we should consider parasite evolution and presenting their argument that all parasites converge on one of six life strategies. In considering the many independent origins of parasitism, they make the case for comparison across taxonomic boundaries in search of consistency in the evolutionary process and limitations on the number of ways of becoming a parasite. If there are ecological constraints on evolution that define adaptive peaks, could there also be genomic constraints? Is it plausible that unrelated parasites will converge in genome structure, content and regulation to meet similar demands for host invasion, complex life cycles and for manipulating host immunity? Poulin and Randhawa (2014) suggest that this is plausible and that, in future, clear trends in parasitic genome architectures might emerge that represent convergent adaptive peaks, the genomic equivalents of the phenotypic strategies used by all parasites.

The origins of parasitic lineages were initially based on comparisons of morphology or ultrastructure with free-living organisms and only relatively recently they have been precisely defined by phylogenetic hypotheses. Genomics provides both the opportunities to test phylogenetic hypotheses and strengthen the robustness of trees through the exhaustive sampling of parasite characters. Rayner and Keeling (2014) review recent insights into the origins of Apicomplexan parasites at two scales: the evolution of the human malarial parasite *Plasmodium falciparum* from among *Laverania* parasites of apes, and the origin of all Apicomplexan parasites from marine photosynthetic ancestors. They demonstrate how better sampling of the biodiversity of these organisms with molecular methods has produced precise hypotheses for the origin of these enigmatic parasites.

In contrast to the single origin of the Apicomplexan parasites, the nematodes include multiple parasitic lineages that have arisen independently from a non-parasitic ancestor. Blaxter and Koutsovoulos (2014) reprise the analysis of the nematode phylogeny in the light of nematode genomics and transcriptomics. While they are apt to emphasize the need of effective free-living comparators for parasitic species, they discuss the evidence from existing genome sequences for themes in nematode parasitism. Horizontal gene transfer and bacterial endosymbiosis are important processes in some lineages but not all, and generally we observe each parasitic lineage modifying the common inheritance in independent ways, for instance with regard to the modification of feeding organs.

Genomics has the potential to describe the evolution of parasitism in terms of genes gained and lost, whereas transcriptomics and proteomics can show how the regulation of conserved genes has evolved. Here a series of four articles by Jackson (2014), Reid (2014), Bird *et al.* (2014) and Zarowiecki and Berriman (2014) explore these changes in detail. The intention is to revisit classical questions of phenotypic reduction and specialization in the light of genome sequencing, but also to ask which mechanisms are important in genomic evolution. Genomic reduction is seen as intuitive among parasites, because, as mentioned previously, of the prominence of reduced phenotypes in some species combined with the received wisdom. Among eukaryotes, this prediction is seemingly confirmed by the Microsporidia, whose genomes are denuded of normally essential biosynthetic pathways (reviewed in Corradi and Slamovits, 2011). However, microsporidians may yet prove to be exceptional and other taxa provide only mild and equivocal support for a general phenomenon (Bird *et al*. 2014; Jackson 2014; Zarowiecki and Berriman, 2014).

Often the most compelling feature of parasite genomes is the multi-copy gene families that encode parasite-specific effector proteins. Genome sequencing has dramatically changed our view of how these genes are arranged and expressed, but also of their origins and evolution. The reviews here emphasize the substantial diversity of parasite-specific gene families in trypanosomatids (Jackson, 2014), Apicomplexa (Reid, 2014), Nematodes (Bird *et al.* 2014) and Platyhelminthes (Zarowiecki and Berriman, 2014). Typically, the very rapid evolution of these gene families results in them being lineage-specific, making it challenging to reconstruct their origins. This is exemplified by the Apicomplexa; Reid (2014) asks whether the very different gene families in plasmodia, piroplasms and coccidians can be reconciled, or if there are themes in their arrangement, despite their lack of homology.

As genome resequencing becomes a standard tool for population genetics of eukaryotic parasites, we are able to examine microevolutionary processes at the genomic level. Chang and Hartl (2014) explore the importance of a complex life cycle in genetic analysis of *P. falciparum* and find that bottlenecks inherent to complex life cycles compromise current methods for detecting a within-host selective advantage, and suggest adjustments for resolving this issue. The abundance of effector genes in most parasite genomes, whether directed at cell invasion or immune modulation, makes it clear that host–parasite co-evolution is a primary determinant of genome content. We would expect parasite genomes to contain the signature of recurrent adaptations and Capewell *et al.* (2014) describe such a situation in African trypanosomes, in which parasite-specific variant surface glycoproteins have been repeatedly recruited as counter-measures to diverse resistance mechanisms evolving in primates.

In the final article, Weedall and Hall (2014) discuss the evidence from genome sequences for sexual reproduction in diverse parasite lineages, which hitherto was often assumed to be absent. Sex is proving to have an intimate relationship with virulence and host range in different pathogens (Bakkeren *et al.* 2012; Bennett and Nielsen, 2012), and so the frequency of sexual reproduction in parasite populations is likely to impact on the evolution of host–parasite interactions, as well as disease epidemiology.

Now that the first flush of parasite genome sequencing is complete it appears that parasite genomes are reduced to some extent relative to free-living organisms. In some cases, this has been considerable, for instance the loss of homeobox genes in tapeworms (Tsai *et al.* 2013); more generally, we must apply some caveats. First, the free-living organisms in question are rarely close relatives (witness the routine use of *Caenorhabditis elegans* as a free-living comparator for all parasitic nematodes) and until genome sequences for appropriate non-parasitic outgroups are available for each parasitic lineage our understanding of genomic change during the evolution of parasitism will be imprecise. Second, the scale of genomic reduction is not comparable to the celebrated Microsporidia, and neither is it indicative of widespread degeneracy. Indeed, is it sensible to label the observed changes ‘reduction’ when there is considerable expansion occurring in the same genomes? Such losses could be within the bounds of normal anagenic change and we need to explore how this rate of gene loss compares to events in purely free-living clades over the same timescales.

In fact, far from a picture of degeneracy, parasite genomes display considerable innovation, chiefly in the form of multi-copy effector gene families and the genomic domains that seem to regulate their expression and diversity. Although such lineage-specific gene family expansions are not unique to parasites, these have the strongest claim to being a theme in parasite genome evolution. Since they are species-specific, our understanding of what these gene families do is patchy, although the needs for diverse surface antigens and for alternative isoforms in different life-cycle stages have clearly driven diversification in many lineages. In this sense, genome sequences are revealing the basis for parasite specialization, and the extent to which interactions with various hosts drove and drive genomic innovation.

A decade of parasite genomics has revealed much of the genetic background to parasite adaptations and also identified purely genomic features, such as position, that are adaptive; for instance the variant antigen expression sites in *Trypanosoma brucei* and *Babesia bovis* (see Jackson, 2014; Reid, 2014). We have seen that the most dynamic features of parasite genomes, those that are typically lineage-specific and which show the greater polymorphism within species and the greatest flexibility in genomic position, are often associated with host interaction and indicate the dominant role of immune selection in directing parasite genome evolution. Given that a signature of recurrent co-evolutionary interactions is evident in extant parasite clades, this may also suggest that a marked increase in evolutionary rate occurs after the adoption of parasitism, as an abiotic environment is exchanged for a host that fights back.

In our search for consistency in parasite genome evolution we find some obvious convergence, antigenic variation for example again and some recurring mechanistic themes (repetition, horizontal gene transfer, paralogous gene family expansion outside of chromosomal cores, rapid turnover of surface antigen genes), but broadly, different lineages have evolved unique solutions to common problems of immunity and secondary metabolism, even among nematode lineages descended from a relatively recent free-living ancestor, and within clades that have a common parasitic ancestor, such as the Apicomplexa or Platyhelminthes.

How we understand the evolution of parasitism was revolutionized by phylogenetics and now by genomics. Still, parasites share the majority of their genes with free-living species and so we must ask how these are used differently. Many fields are rapidly applying increasingly data-rich ‘omic’ methods to study parasite physiology on a whole cell scale. When we next revisit reduction and innovation during the origin of parasites, this may concern the networks of interacting RNA and protein species that dynamically control parasite development and physiology.
